# The intellectual landscape of cognitive impairment in type 2 diabetes: knowledge structure, research focuses and rising trends

**DOI:** 10.3389/fendo.2026.1809245

**Published:** 2026-04-01

**Authors:** Hui Han, Libo Hou, Jinghua Lu, Hao Sun, Wenwen Ning, Yue Liang, Yujie Gu, Huichao Yin, Qiang Gao

**Affiliations:** Department of Rehabilitation Medicine, The Affiliated Taian City Central Hospital of Qingdao University, Taian, China

**Keywords:** bibliometric analysis, cognitive dysfunction, development trends, research hotspots, type 2 diabetes

## Abstract

**Background:**

Type 2 diabetes mellitus (T2DM) is prevalent worldwide, with cognitive dysfunction emerging as a significant complication. Despite extensive research into its pathological mechanisms and clinical management, the knowledge structure, research priorities, and developmental trends within this field remain unsystematically integrated.

**Methods:**

To ensure comprehensive coverage and compatibility with bibliometric analysis tools, relevant publications on type 2 diabetes mellitus (T2DM) and cognitive dysfunction were primarily retrieved from the Web of Science Core Collection (WOSCC) database from its inception to November 5, 2025. Searches were also conducted in PubMed and Scopus to verify completeness, but the final dataset was derived mainly from WOSCC due to its optimal export format for CiteSpace (plain text with full records and cited references) and high overlap after deduplication. Employing bibliometric methods and tools such as CiteSpace (version 6.4 Advanced) and SCImago Graphica (version 1.0.39), we conducted visual analyses of publication trends, country/region collaboration, institutional distribution, author contributions, journal impact, co-cited references, and keywords.

**Results:**

A total of 1,752 publications were included. Annual publication volume exhibited a marked upward trend, accelerating particularly after 2018. China, the United States, and the United Kingdom were the primary contributing nations, with close inter-institutional collaboration. High-frequency keywords included ‘type 2 diabetes mellitus’, ‘Alzheimer’s disease’, and ‘cognitive impairment’. Research focus has shifted from early risk factors to microscopic levels, including neuroimaging, gut microbiota, and molecular mechanisms.

**Conclusion:**

Research on cognitive dysfunction in T2DM exhibits multidisciplinary characteristics, balancing fundamental research with clinical translation. Future efforts should enhance multidimensional integration of mechanism studies to advance early screening and personalised treatment strategies.

## 1 Introduction

T2DM is a common metabolic disease. In 2022, the number of people with diabetes reached 828 million, among whom 95% were T2DM (1). The number of people with T2DM is expected to reach 1.31 billion by 2050 (2). With the wide prevalence of T2DM, the cognitive dysfunction that accompanies it (including mild cognitive impairment and dementia) has received increasing attention and is regarded as an essential complication (3). Cognitive dysfunction has a profound adverse impact on the disease management and individual quality of life of patients with T2DM. It is associated with the development of other complications, exacerbating the patient’s condition (4). Therefore, guidelines for T2DM recommend screening elderly patients for cognitive dysfunction and developing personalised treatment plans for this population to enhance therapeutic efficacy and adherence.

Cognitive dysfunction encompasses severe disorders (such as dementia and severe neurocognitive disorders) to mild cognitive impairment and mild neurocognitive disorders (5). Although the performance levels of cognitive dysfunction in diabetes do not fall markedly within the abnormal range, they are typically approximately 0.3 to 0.5 standard deviations lower than those of non-diabetic individuals. A study involving 26,137 patients demonstrated that diabetic patients exhibit poorer performance in verbal and visual memory, attention and concentration, processing speed, executive function, and motor control (6). Compared with non-diabetic individuals, those with diabetes exhibit a substantially increased risk of developing mild cognitive dysfunction (7). Risk factors contributing to cognitive dysfunction in T2DM include age, hyperglycaemia, and unhealthy lifestyle habits. Cognitive function progressively declines with advancing age, with impairment more pronounced in elderly patients (8). This phenomenon is associated with oxidative stress, fluctuating blood glucose levels, insulin resistance, and the accumulation of amyloid-β protein deposits within the brain (9). Chronic hyperglycaemia accelerates the production of advanced glycation end-products (AGEs), which accumulate in blood and tissues (10). Hyperglycaemia-mediated AGE production, alongside oxidative stress, is recognised as a factor contributing to neuronal degeneration and vascular endothelial damage, thereby impairing cognitive function (11). Research indicates that unhealthy lifestyles and physical inactivity exacerbate cognitive decline (12, 13).

Given the widespread incidence and adverse effects of cognitive dysfunction in T2DM, scholars have conducted extensive research to explore the disease’s mechanisms and clinical management strategies. Studies reveal that insulin regulates cortical activity and cerebral metabolism, playing a crucial role in cognition by controlling the production of the neurotransmitter acetylcholine (14), offering a novel perspective for investigating the pathological mechanisms of this condition. Pro-inflammatory factors, such as tumour necrosis factor and interleukins, are elevated in T2DM patients, exacerbating neuronal damage and accelerating cognitive decline (15). Concurrently, dysfunction of the hypothalamic-pituitary-adrenal (HPA) axis, elevated cortisol levels, and vascular complications in T2DM patients also contribute to cognitive dysfunction (16–18). Despite extensive research and positive findings, systematic integration of the current research landscape, key discoveries, and future trends remains lacking. This study therefore employs bibliometric analysis to review and analyse the literature systematically, summarising the field’s knowledge structure, research status, and emerging issues. It further explores future research directions to provide robust evidence for advancing this area of inquiry.

## 2 Materials and methods

### 2.1 Data sources and search strategy

To ensure comprehensive literature coverage while maintaining compatibility with bibliometric visualization tools, we searched three major databases: the Web of Science Core Collection (WOSCC), PubMed, and Scopus, from database inception to November 5, 2025.

Searches were performed using standardized terms related to Type 2 Diabetes Mellitus and Cognitive Dysfunction (see **Supplementary Table 1**). Specifically: WOSCC: Topic (TS) field search, PubMed: Title/Abstract search, Scopus: TITLE-ABS-KEY search.

All retrieved records were exported in plain text format and imported into EndNote X9 for deduplication and screening.

### 2.2 Data processing and analysis

**Figure 1:**
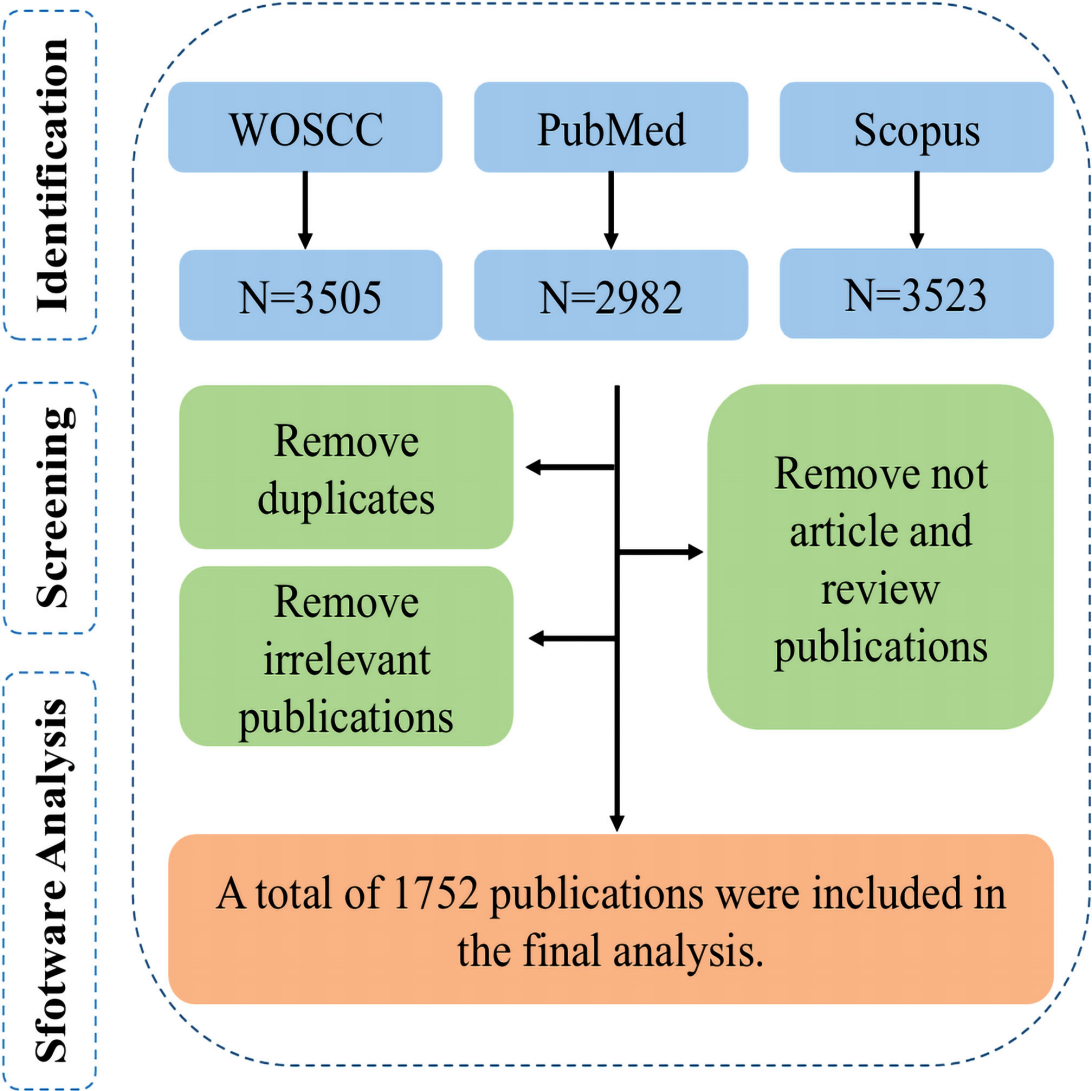
Publication screening process.

Automatic deduplication was performed based on DOI, title, authors, and publication year, followed by manual review of titles and abstracts to remove remaining duplicates and irrelevant records. Due to the high overlap among databases and the superior compatibility of WOSCC export format with CiteSpace, the final dataset of 1,752 eligible publications (1,328 articles and 424 reviews, restricted to English language and article/review types) was primarily derived from WOSCC. **Figure 1** illustrates the specific screening process.

All citation-based metrics, including betweenness centrality, citation bursts, co-citation networks, and keyword correlations, were calculated exclusively using the WOSCC-derived dataset in CiteSpace (v. 6.4 Advanced). This choice ensures the integrity of citation linkage data, as CiteSpace is optimized for Web of Science export formats, and supplementary data from PubMed and Scopus were not incorporated into the network analyses due to format compatibility limitations.

Microsoft Excel 2019, CiteSpace (v. 6.4), and SCImagoGraphica (v. 1.0.39) were used for data analysis. Microsoft Excel 2019 was used to analyse publication trends; CiteSpace (v.6.4) was employed for analysing institutional co-occurrence views, author-publication and author-citation co-occurrence views, journal citation co-occurrence views, journal double-map overlays, co-cited document co-occurrence views, and keyword correlation analysis; SCImagoGraphica (v.1.0.39) was utilised for country/region distribution and collaboration analysis, institutional collaboration analysis, and author collaboration analysis.

The parameters of CiteSpace (v.6.4 Advanced) software were set as follows: Time Span: 2003-2025; Slice Length: 1 year; Selection Criteria: g index(k=25); Pruning: pathfinder, pruning sliced networks. After the setup was completed, authors, institutions, references, cited authors, cited journals, and keywords were selected as network nodes for visualization and analysis, respectively. The main parameters of SCImago Graphica (v.1.0.39) software are set as follows: Size: frequency; Color: clusters; Label: choose according to the content of the analysis, Use the same color as marks; Layout: Circular; Edges: Use the same color as marks.

The Ethics Committee waived ethical approval as the data originated from public databases and did not involve human or animal subjects.

## 3 Results

### 3.1 Trend in publication growth

**Figure 2:**
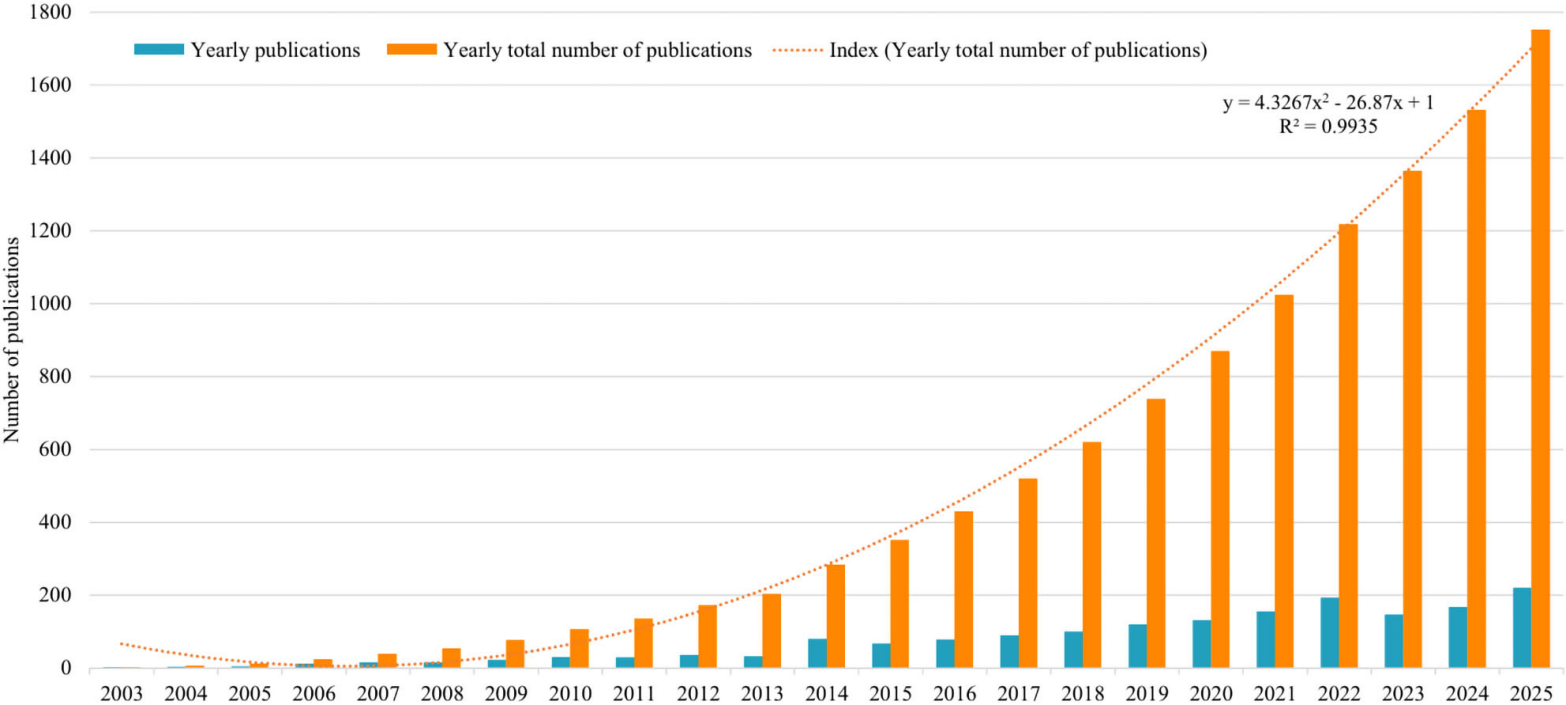
Publication trend chart.

**Figure 2** illustrates the annual publication volume. The 1,752 documents analysed in this study were published between 2003 and 2025, with publication numbers rising steadily, peaking in 2025. Publication numbers experienced three significant milestones: surpassing 50 publications for the first time in 2014, reaching 100 publications in 2018, and achieving rapid growth thereafter. By 5 November 2025, the annual publication count had already reached 220, with projections indicating a total of 260 publications for the year. Despite a slight dip in output during 2023-2024, publication levels remained high, indicating sustained scholarly interest in this research domain. Analysis of the annual publication trend line reveals a quadratic coefficient of 4.3267, indicating accelerated growth over time—that is, the rate of increase is progressively rising. The linear coefficient is -26.87, indicating slower growth during the initial phase. The R² value, ranging from 0 to 1, measures the fit of the trend line to the actual data. Here, R² = 0.9935 indicates that the quadratic model explains 99.35% of the variation in the data, indicating a very good fit to the observed data (explaining 99.35% of the variance in publication volume). However, high R² in time-series trend fitting primarily reflects descriptive goodness-of-fit and does not necessarily imply strong predictive validity or guarantee future trends. Based on the analysis of annual publication volume and total annual publication volume, the observed pattern suggests a continuing upward trend in research interest, though future volumes may be influenced by various external factors.

### 3.2 Distribution and cooperation by country/region

**Figure 3:**
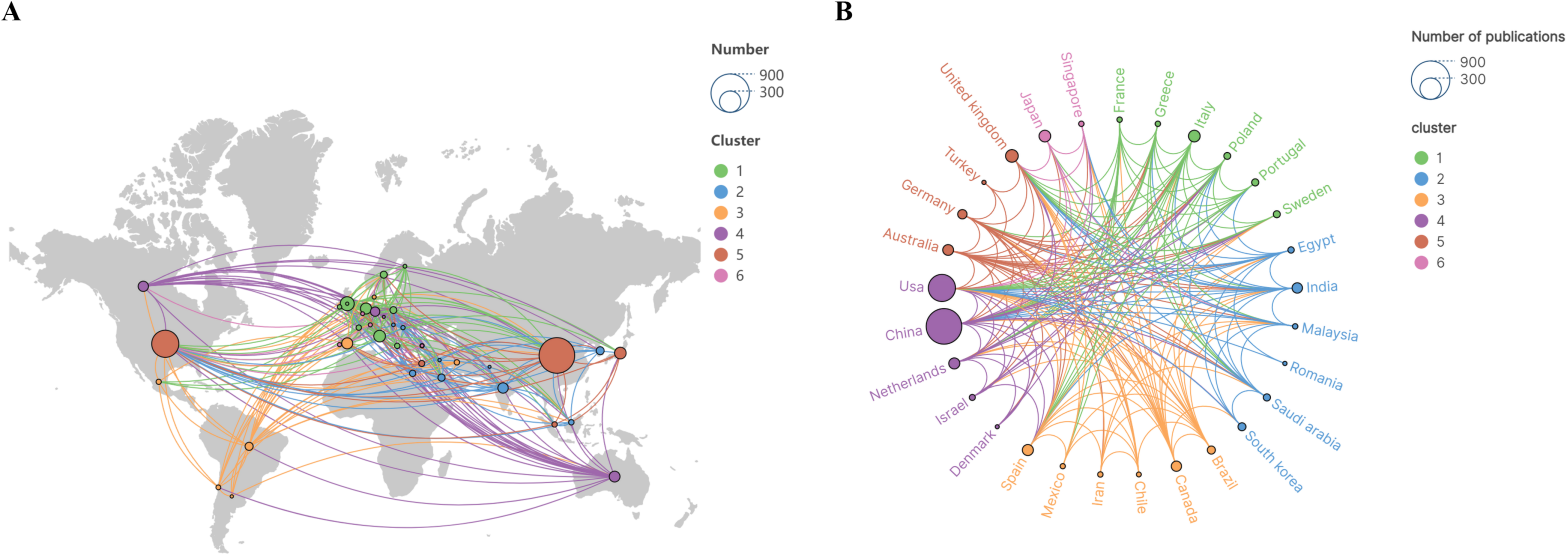
**(A)** Global distribution of publications. **(B)** Country/region collaboration.

A total of 350 countries or regions contributed publications on this subject. **Table 1** presents the top 15 countries by publication volume, each with at least 27 publications. **Figure 3A** illustrates the geographical distribution of these countries. Research citations related to this topic are widely prevalent globally. In terms of publication volume, China, the United States, and the United Kingdom are the primary contributors to this research theme, significantly outpacing other countries or regions. Regarding betweenness centrality in the country/region collaboration network (calculated using CiteSpace), the United States, the United Kingdom, and Germany hold leading positions. Betweenness centrality measures the extent to which a node (here, a country) lies on the shortest paths connecting other nodes in the network, reflecting its bridging or mediating role in international collaborations and knowledge flow (19). High centrality indicates that these countries serve as pivotal connectors between different research communities or clusters, facilitating interdisciplinary or cross-regional interactions, rather than directly denoting research quality or pioneering status per se.

**Table 1:** Top 15 countries/regions by publication volume.

Rank	Count	Centrality	Country
1	699	0.06	CHINA
2	406	0.46	USA
3	110	0.22	UNITED KINGDOM
4	75	0.05	ITALY
5	75	0	JAPAN
6	67	0.06	SPAIN
7	67	0.09	NETHERLANDS
8	64	0.14	AUSTRALIA
9	59	0.05	INDIA
10	58	0.13	CANADA
11	48	0.21	GERMANY
12	36	0.11	BRAZIL
13	35	0.01	SOUTH KOREA
14	28	0.03	SAUDI ARABIA
15	27	0.01	SWEDEN

**Figure 3B** illustrates collaborative relationships among leading publishing nations or regions, which cluster into six distinct groups, indicating extensive international cooperation. Notably, China, the United States, the United Kingdom, and the NETHERLANDS each maintain stable and prominent collaborative ties with other nations. This underscores the vital role of international collaboration in advancing research within this thematic domain.

### 3.3 Institutional analysis

**Figure 4:**
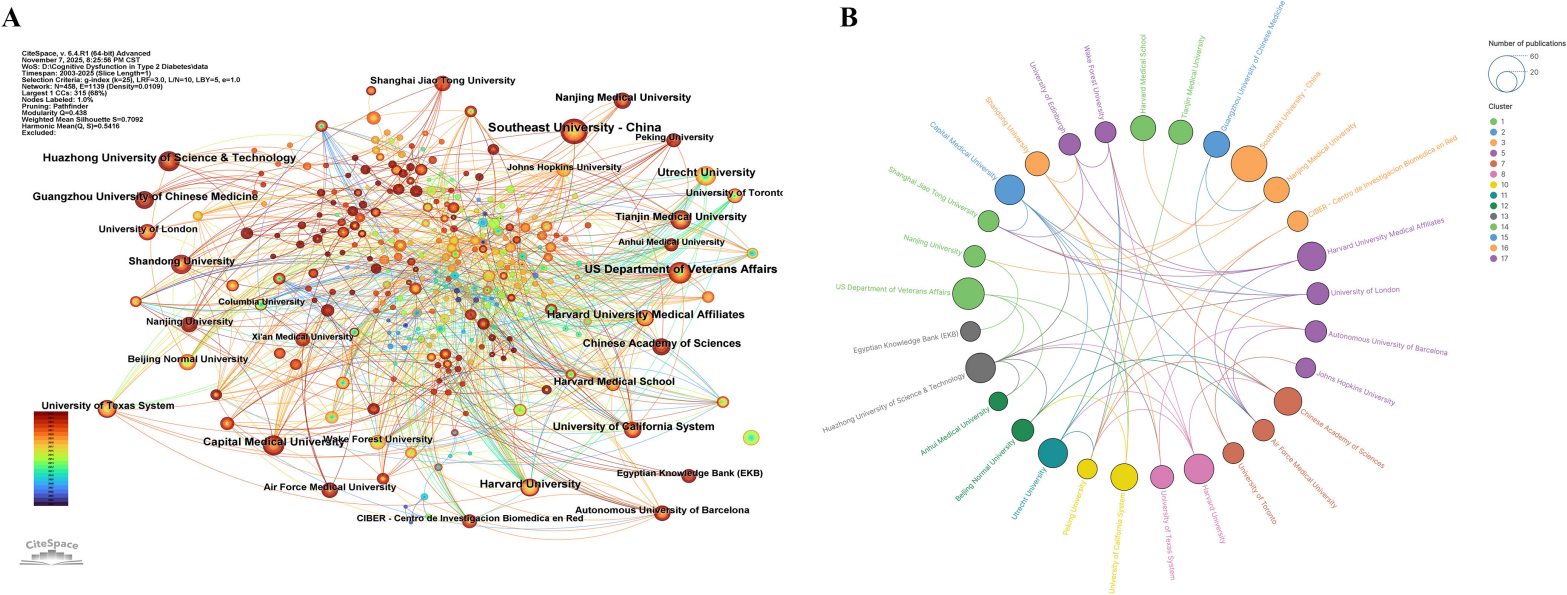
**(A)** Co-occurrence network view of institutions. **(B)** Collaborative relationship network view of major institutions.

A total of 6,499 institutions contributed publications on this topic, with **Table 2** presenting the top 10 institutions by publication volume. All 10 institutions published no fewer than 29 papers, with the top two—Southeast University (China) and the US Department of Veterans Affairs—contributing 55 and 43 papers respectively. In terms of centrality, Capital Medical University ranked first at 0.1. The three most prolific institutions all achieved a centrality of 0.06, while Utrecht University also attained 0.06, placing it among the leaders. **Figure 4A** presents a co-occurrence analysis network view of these institutions, while **Figure 4B** displays a collaboration network view of the principal institutions, grouped into 17 collaborative clusters. Organisations within each cluster exhibit close collaborative ties, while distinct cooperative relationships also exist between clusters. Huazhong University of Science & Technology, Capital Medical University, Wake Forest University, and Air Force Medical University demonstrate particularly significant collaboration with other institutions.

**Table 2:** Top 10 institutions by publication volume.

Rank	Institutions	Count	Centrality	Total link strength
1	Southeast University – China	55	0.06	19
2	US Department of Veterans Affairs	43	0.06	18
3	Harvard University	38	0.06	20
4	Huazhong University of Science & Technology	38	0.04	32
5	Capital Medical University	38	0.1	27
6	Utrecht University	37	0.06	26
7	Harvard University Medical Affiliates	35	0.04	42
8	Chinese Academy of Sciences	33	0.03	32
9	University of California System	31	0.02	15
10	Guangzhou University of Chinese Medicine	29	0.03	21

### 3.4 Author’s analyses and collaborative relationships

**Figure 5:**
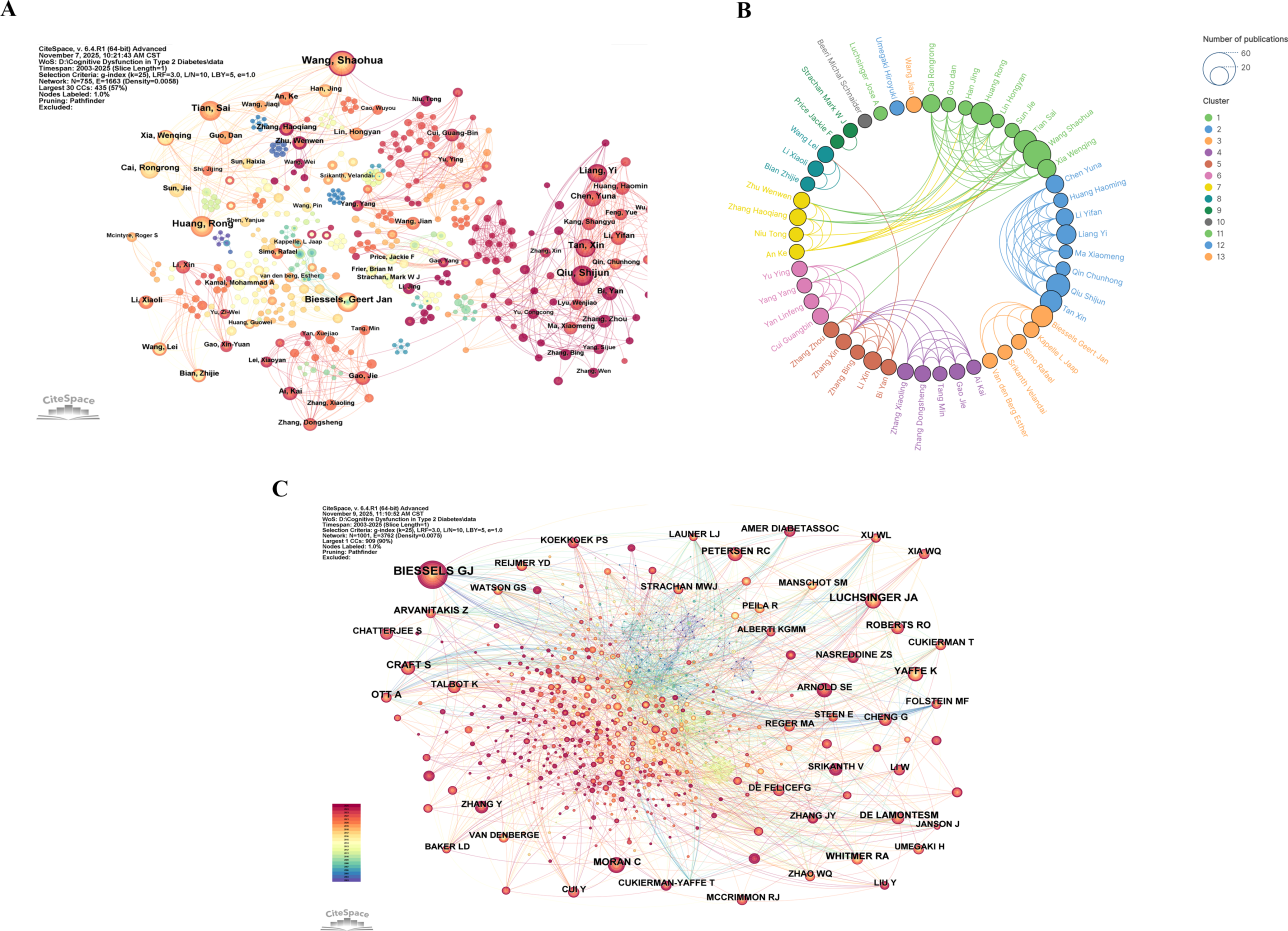
**(A)** Network view of published authors. **(B)** Collaboration network view of published authors. **(C)** Network view of cited authors.

A total of 9,279 authors contributed to publications within this field. **Table 3** presents the top ten authors by number of publications, each having authored no fewer than 14 papers. Wang, Shaohua, published the most papers (36), significantly exceeding the other contributors. Qiu, Shijun, ranks second with 27 publications and a centrality of 0.01, leading among the top ten scholars. Considering total link strength, Wang, Shaohua, Tian, Sai, and Huang, Rong occupy the top three positions, indicating substantial collaborative ties with other researchers. **Figure 5A** presents a network view of publishing authors, while **Figure 5B** illustrates collaboration relationships among core publishing authors. These authors form relatively stable collaborative clusters, though interactions remain confined within clusters, with limited inter-cluster collaboration.

**Table 3:** Top 10 authors by publication volume.

Rank	Authors	Count	Centrality	Total link strength
1	Wang, Shaohua	36	0	166
2	Qiu, Shijun	27	0.01	107
3	Huang, Rong	26	0	130
4	Biessels, Geert Jan	23	0	28
5	Tian, Sai	23	0	133
6	Tan, Xin	21	0	107
7	Liang, Yi	20	0	90
8	Cai, Rongrong	16	0	86
9	Chen, Yuna	15	0	86
10	Xia, Wenqing	14	0	52

**Table 4** presents the top 10 most highly cited authors, each with at least 165 citations. Among them, Geert Jan Biessels from the UMC Utrecht Brain Center holds the highest citation count at 693. Regarding centrality, Anna Ott from the Centre for Statistical Genetics in West Orange ranks first among the 10 scholars, with a centrality of 0.08. **Figure 5C** presents a network visualisation of the highly cited authors.

**Table 4:** Top 10 scholars by number of publications cited.

Rank	Cited author	Citations	Centrality	Institution
1	Geert Jan Biessels	693	0.11	UMC Utrecht Brain Center
2	José A Luchsinger	310	0.05	Columbia University Irving Medical Center
3	Suzanne Craft	266	0.07	Wake Forest School of Medicine
4	Kristine Yaffe	236	0.02	Johns Hopkins Bloomberg School of Public Health
5	Chris Moran	225	0.01	Monash University
6	Anna Ott	208	0.08	Center of Statistical Genetics, West Orange
7	Ronald C Petersen	192	0.03	Mayo Clinic, Rochester, Minnesota, USA.
8	Michael J LaMonte	185	0.05	University at Buffalo - SUNY
9	Zoe Arvanitakis	171	0.06	Rush University Medical Center
10	Rosebud O Roberts	165	0.02	Mayo Clinic College of Medicine

### 3.5 Analysis of publishing journals and cited journals

A total of 542 journals published works related to this subject, with **Table 5** presenting the top 10 journals by publication volume. These ten journals collectively published 356 articles, accounting for 20.32% of all publications. Among them, JOURNAL OF ALZHEIMERS DISEASE (Q2/3.1) published the highest number of articles (80 articles/4.6%), followed by FRONTIERS IN AGING NEUROSCIENCE (Q1/4.5; 50/2.85%) and FRONTIERS IN NEUROSCIENCE (Q2/3.2; 41/2.34%). These journals predominantly focus on diabetes and neuroscience, aligning closely with research in this field.

**Table 5:** Top 10 journals by publication volume.

Rank	Journal	Count	Division/IF
1	JOURNAL OF ALZHEIMERS DISEASE	80	Q2/3.1
2	FRONTIERS IN AGING NEUROSCIENCE	50	Q1/4.5
3	FRONTIERS IN NEUROSCIENCE	41	Q2/3.2
4	FRONTIERS IN ENDOCRINOLOGY	34	Q1/4.6
5	INTERNATIONAL JOURNAL OF MOLECULAR SCIENCES	33	Q1/4.9
6	DIABETES RESEARCH AND CLINICAL PRACTICE	29	Q1/7.4
7	PLOS ONE	28	Q2/2.6
8	JOURNAL OF DIABETES AND ITS COMPLICATIONS	23	Q2/3.1
9	DIABETES CARE	19	Q1/16.6
10	DIABETOLOGIA	19	Q1/10.2

**Figure 6:**
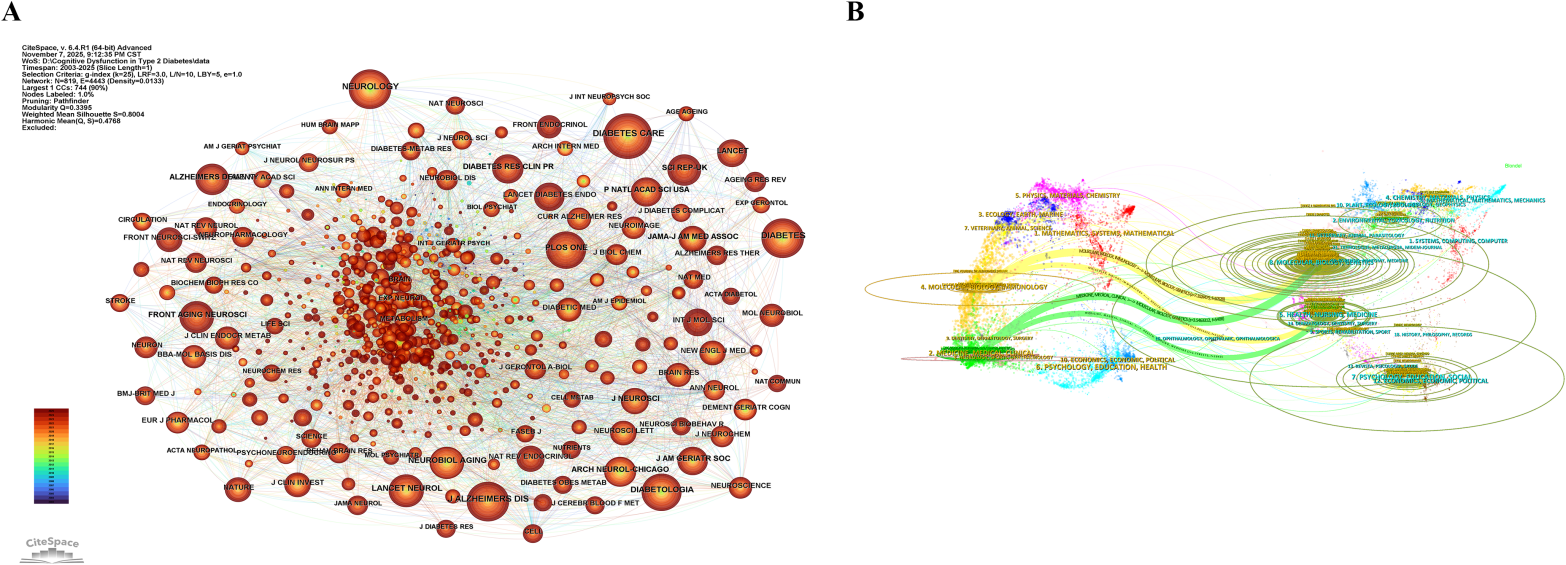
**(A)** Network view of cited journals. **(B)** Overlay analysis view of journal dual maps.

We analysed the cited journals, with **Table 6** presenting the top 10 journals by citation frequency. **Figure 6A** displays a network analysis view of the cited journals. DIABETES CARE (Q1/16.6; cited 1257 times) ranks as the most frequently cited journal, followed by J ALZHEIMERS DIS (Q2/3.1; cited 1028 times) and NEUROLOGY (Q1/9; cited 1009 times). DIABETES (Q1/7.5) also exceeded 1,000 citations, reaching 1,003. All ten journals are in the Q1/Q2 quartiles and generally have high impact factors, indicating the high quality and significant reference value of the research published therein.

**Table 6:** Top 10 Journals by cited times.

Rank	Count	Cited journal	Division/IF
1	1257	DIABETES CARE	Q1/16.6
2	1028	J ALZHEIMERS DIS	Q2/3.1
3	1009	NEUROLOGY	Q1/9
4	1003	DIABETES	Q1/7.5
5	919	DIABETOLOGIA	Q1/10.2
6	856	PLOS ONE	Q2/2.6
7	754	LANCET NEUROL	Q1/45.5
8	700	NEUROBIOL AGING	Q2/3.5
9	652	ARCH NEUROL-CHICAGO	/
10	636	ALZHEIMERS DEMENT	Q1/11.1

In **Figure 6B**, the journal’s dual-mapping overlay analysis view illustrates the distribution of topics. Labels denote the disciplinary themes covered, while coloured paths represent citation relationships. The analysis reveals four prominent citation pathways. It is evident that publications from journals under the themes ‘MOLECULAR, BIOLOGY, GENETICS’ and ‘HEALTH, NURSING, MEDICINE’ are frequently cited by journals under ‘MOLECULAR, BIOLOGY, IMMUNOLOGY’ and ‘MEDICINE, MEDICAL, CLINICAL’. The divergent and intersecting citation pathways among these four themes indicate that research on cognitive dysfunction in T2DM spans a broad range of disciplines and exhibits diverse characteristics.

### 3.6 Co-cited references analysis

**Figure 7:**
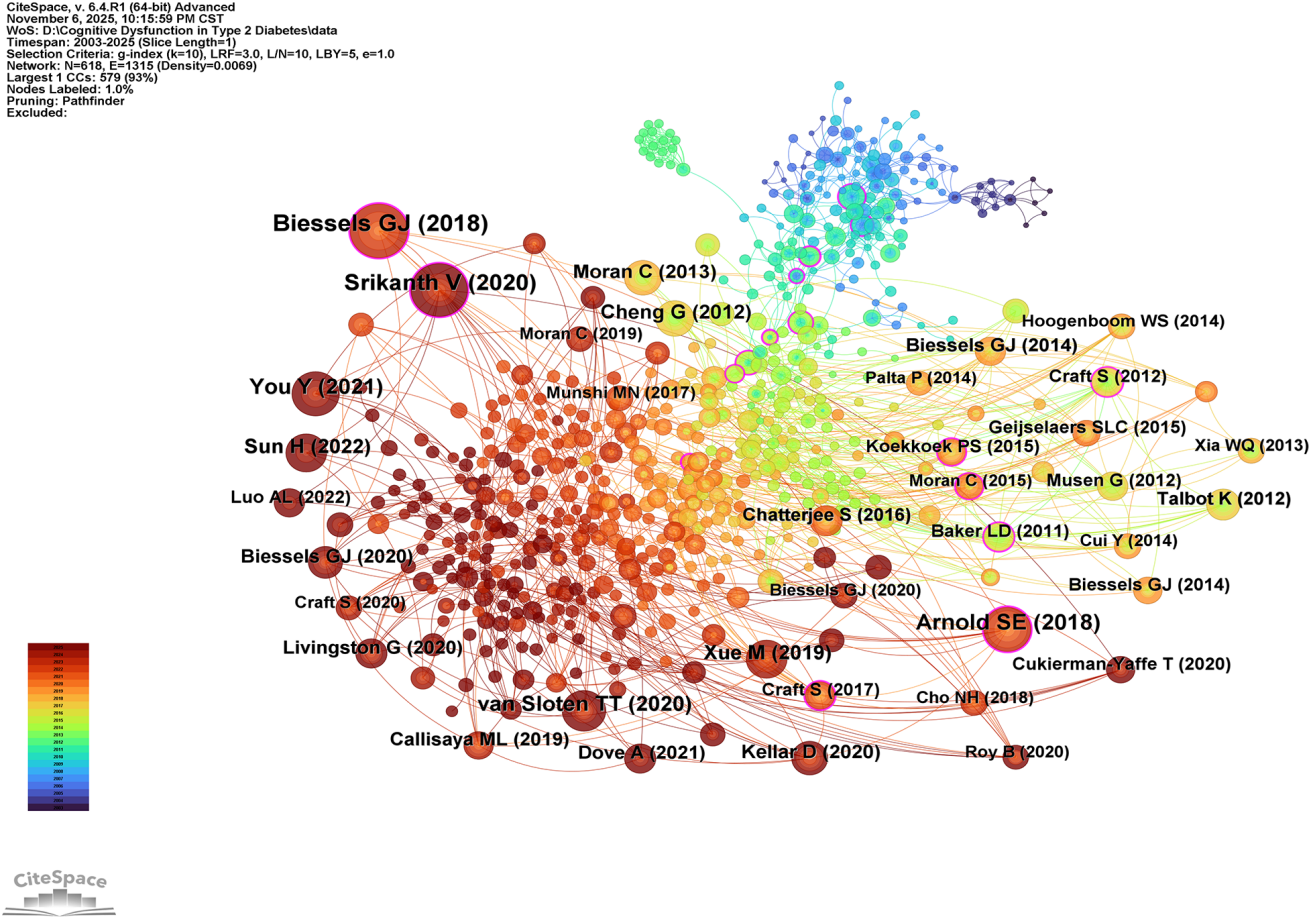
Network analysis view of co-cited references.

When multiple publications are cited repeatedly by different works, co-citation relationships emerge. These relationships are often used to assess the degree of association between disparate publications. **Table 7** presents the top ten most commonly cited references, whilst **Figure 7** displays a network visualisation of the co-occurrence analysis for these references. The most frequently cited publication is ‘Cognitive decline and dementia in diabetes mellitus: mechanisms and clinical implications’ by Biessels GJ, published in NAT REV ENDOCRINOL in 2018. This is followed by Srikanth V’s 2020 paper in The Lancet Diabetes & Endocrinology, ‘Type 2 diabetes and cognitive dysfunction—towards effective management of both comorbidities’, and Arnold SE’s 2018 paper in Nature Reviews Neurology, ‘Brain insulin resistance in type 2 diabetes and Alzheimer disease: concepts and conundrums’. These publications all focus on the mechanisms and clinical management of cognitive dysfunction in T2DM.

**Table 7:** Top 10 most cited references.

Rank	Count	Centrality	Year	Cited reference
1	113	0.17	2018	Biessels GJ, 2018, NAT REV ENDOCRINOL, V14, P591, DOI 10.1038/s41574-018-0048-7
2	109	0.13	2020	Srikanth V, 2020, LANCET DIABETES ENDO, V8, P535, DOI 10.1016/S2213-8587(20)30118-2
3	83	0.14	2018	Arnold SE, 2018, NAT REV NEUROL, V14, P168, DOI 10.1038/nrneurol.2017.185
4	80	0.06	2021	You Y, 2021, ACTA DIABETOL, V58, P671, DOI 10.1007/s00592-020-01648-9
5	67	0.03	2020	van Sloten TT, 2020, LANCET DIABETES ENDO, V8, P325, DOI 10.1016/S2213-8587(19)30405-X
6	58	0.01	2022	Sun H, 2022, DIABETES RES CLIN PR, V183, P0, DOI 10.1016/j.diabres.2021.109119
7	58	0.03	2019	Xue M, 2019, AGEING RES REV, V55, P0, DOI 10.1016/j.arr.2019.100944
8	51	0.02	2012	Cheng G, 2012, INTERN MED J, V42, P484, DOI 10.1111/j.1445-5994.2012.02758.x
9	49	0.08	2020	Kellar D, 2020, LANCET NEUROL, V19, P758, DOI 10.1016/S1474-4422(20)30231-3
10	48	0.01	2013	Moran C, 2013, DIABETES CARE, V36, P4036, DOI 10.2337/dc13-0143

### 3.7 Keyword analysis

#### 3.7.1 Keywords co-occurrence analysis

**Figure 8:**
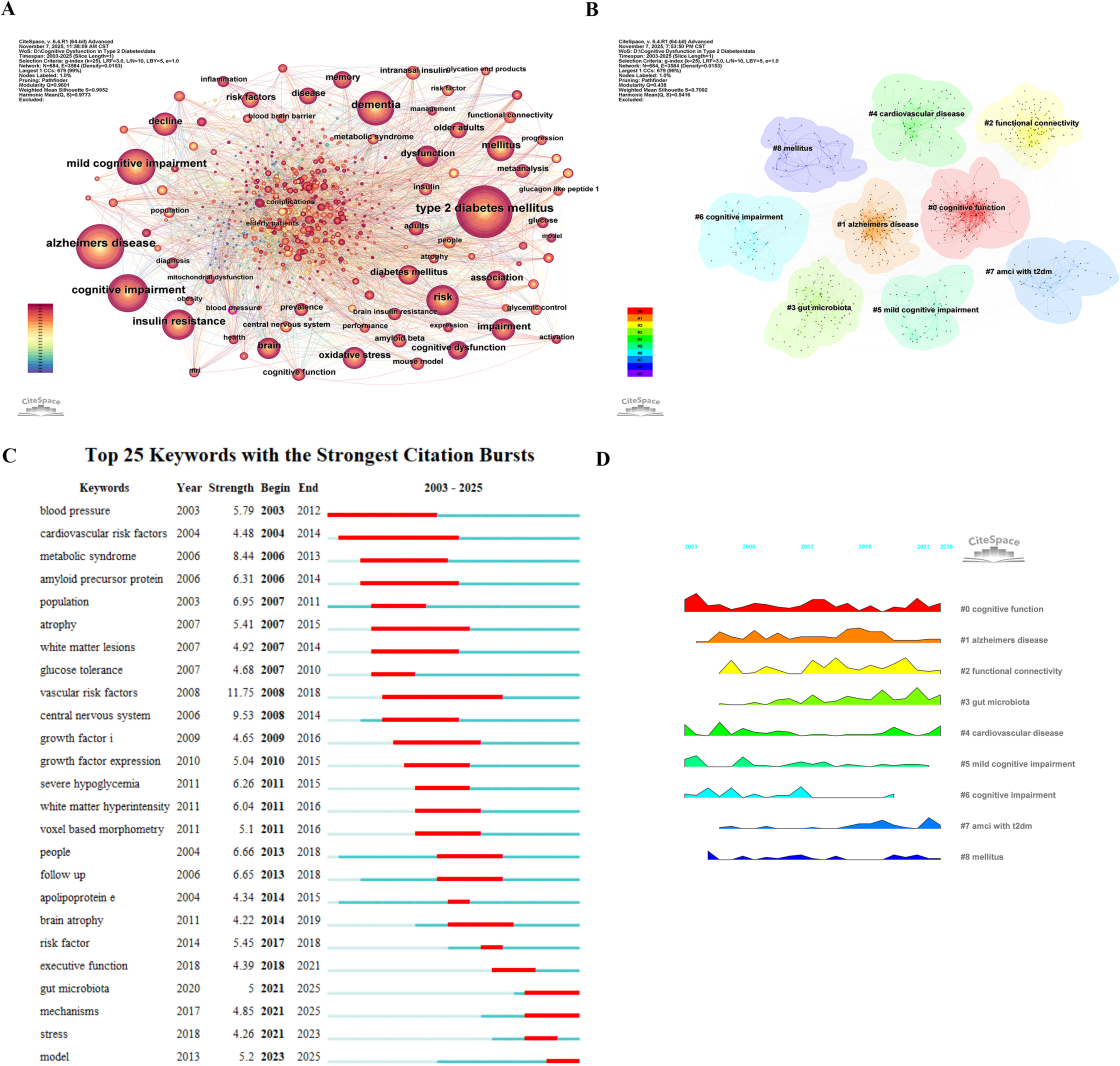
**(A)** Co-occurrence network view of keywords. **(B)** Keywords clustering analysis network view. **(C)** Keywords salience analysis view. **(D)** Keywords landscape analysis view.

Among the 1,752 publications, 683 keywords were identified, reflecting the focal points and cohesive themes within this research domain. **Table 8** presents the 15 most frequently occurring keywords, with type 2 diabetes mellitus ranking first at 960 occurrences, followed by Alzheimer’s disease at 742 occurrences. Cognitive impairment, dementia, and mild cognitive impairment followed closely, appearing 524, 486, and 468 times respectively. Other high-frequency keywords included brain, oxidative stress, dysfunction, association, and risk factors. These keywords encompass disease symptoms and associated mechanisms of action, reflecting the focus of scholarly attention. The co-occurrence network diagram of keywords is shown in **Figure 8A**, where node size reflects keyword frequency.

**Table 8:** The 15 most frequently occurring keywords.

Rank	Count	Centrality	Keywords
1	960	0.02	type 2 diabetes mellitus
2	742	0.01	alzheimers disease
3	524	0.03	cognitive impairment
4	486	0.03	dementia
5	468	0.08	mild cognitive impairment
6	369	0.02	insulin resistance
7	348	0.02	risk
8	266	0.05	mellitus
9	216	0.02	decline
10	211	0.02	impairment
11	198	0.06	brain
12	192	0.03	oxidative stress
13	187	0.04	dysfunction
14	166	0.04	association
15	155	0.03	risk factors

#### 3.7.2 Keywords cluster analysis

**Figure 8B** presents a network view of the clustered keywords, comprising 684 nodes and 3,584 links. Analysis indicates Q = 0.438 > 0.3 and S = 0.7029 > 0.7, confirming a stable cluster structure and reliable results. These keywords cluster into nine groups, with “cognitive function” forming the largest cluster. This indicates that research in this domain primarily focuses on the core symptoms of the disease, with restoring cognitive function being the paramount research objective. The second-largest cluster is “Alzheimer’s disease”, suggesting scholars also examine the link between T2DM and this condition. Considering other clusters, “cardiovascular disease” and cognitive dysfunction in T2DM are closely related and attract scholarly attention. However, overall, scholars remain concentrated on cognition-related research, encompassing the classification of cognitive impairment (#5: mild cognitive impairment; #6: cognitive impairment; #7: amci with t2dm) and associated therapeutic pathways and mechanisms of action (#2: functional connectivity and #3: gut microbiota).

#### 3.7.3 Keywords burst analysis

To further explore shifts in research focus, keyword burst analysis identified 25 key terms with the strongest citation bursts (**Figure 8C**). Burst strength quantifies the intensity of emergence, with higher values indicating stronger surges in scholarly attention. Early bursts (2003–2010) were dominated by macro-level risks, such as “blood pressure” (strength 5.79, 2003–2012), “cardiovascular risk factors” (4.44, 2004–2014), and “amyloid precursor protein” (6.31, 2006–2013), reflecting initial emphasis on vascular and AD-related pathologies. Mid-phase (2011–2018) showed deepening into mechanisms and techniques, with high-strength bursts like “central nervous system” (9.53, 2008–2014), “vascular risk factors” (11.75, highest overall, 2008–2018), and “voxel-based morphometry” (implied in related terms). Recent phase (2018–2025) shifted to microscopic/systemic perspectives, evidenced by persistent/recent bursts in “gut microbiota” (persistent to 2025, aligning with Cluster #3), “mechanisms” (strength 4.85, 2017–2025), “stress” (4.26, 2018–2023), and “model” (strength 5.2, 2021–2025 or recent). These recent bursts (strength >4) collectively indicate a quantitative transition toward integrative and mechanistic studies, with average burst strength in Phase Three (~4.8–5.2) comparable to or exceeding early vascular-focused terms, underscoring sustained and deepening interest.

#### 3.7.4 Keywords landscape analysis

The keyword landscape (**Figure 8D**) reveals a multi-dimensional knowledge system. Temporal spans and peak phases quantify thematic evolution: persistent clusters (#0 cognitive function, #1 Alzheimer’s disease, #4 cardiovascular disease, #5 mild cognitive impairment) span the entire 2003–2025 period, indicating foundational continuity. Bridging clusters (#2 functional connectivity, #3 gut microbiota) show recent peaks, consistent with burst data. The emergence of “model” (burst strength 5.2, recent) as a late-emerging keyword suggests growing efforts to develop integrated theoretical or computational models for synthesizing complex pathological pathways, supported by its high burst intensity relative to prior terms and alignment with trends toward mechanistic integration in Phase Three. This interpretation is grounded in the burst strength and temporal patterns, rather than speculation.

## 4 Discussion

### 4.1 Research overview on cognitive dysfunction in T2DM

This study systematically reviewed the research trajectory of cognitive dysfunction in T2DM based on 1,752 relevant publications between 2003 and 2025. Findings indicate a pronounced upward trend in research activity within this field, with publication volumes rising significantly after 2018 and peaking in 2025. This growth reflects increasing global attention to cognitive health in T2DM patients and indicates the field remains in a phase of rapid development.

In terms of country/region distribution, China, the United States, and the United Kingdom are the primary contributors. These three nations not only lead in publication volume but also exhibit high centrality, indicating significant research influence. Collaborative networks further reveal robust multinational partnerships, with particularly prominent cooperation between China, the United States, the United Kingdom, and the Netherlands, underscoring the field’s distinctly international character. China exhibited the highest publication output and strong betweenness centrality in the collaboration network, reflecting its rapid growth in T2DM-cognitive dysfunction research. However, the restriction to English-language publications and primary reliance on WOSCC may have influenced geographic representation, potentially amplifying the visibility of Chinese contributions (due to increasing English publications from China in WoS-indexed journals) while underrepresenting research from non-English-speaking regions. This language and database bias should be considered when interpreting global trends.

Institutional collaboration and author distribution indicate that universities and medical institutions form the research backbone. Institutions such as Southeast University – China, the US Department of Veterans Affairs, and Harvard University exhibit high publication volumes and collaborative intensity within this domain. Among authors, scholars such as Wang, Shaohua and Qiu, Shijun demonstrate active participation in both publication output and collaborative networks. Meanwhile, the high citation frequency of Biessels, G. J. reflects his profound influence in mechanism and clinical translation research.

Journal analysis indicates that research outcomes predominantly appear in high-impact journals at the diabetes-neuroscience interface, such as DIABETES CARE, JOURNAL OF ALZHEIMERS DISEASE, and NEUROLOGY. Overlay analysis further reveals cross-thematic research, with frequent citation pathways between basic research (e.g., molecular biology, genetics) and clinical medicine, underscoring the field’s pronounced multidisciplinary convergence.

Co-citation analysis further confirmed the knowledge foundations of this field. The most frequently cited literature predominantly focused on the pathogenesis and clinical management strategies for cognitive dysfunction in T2DM. Notably, the reviews by Biessels, G. J (20).and Srikanth, V (21).systematically summarised the pathological links between diabetes and cognitive decline, providing comprehensive theoretical underpinnings for research in this domain.

### 4.2 Research hotspots and future trends in cognitive dysfunction in T2DM

Keywords reflect the interrelationships among the various themes addressed in the literature, serving as the central summaries of an article. Analysing keywords facilitates the identification of research hotspots within the field. Analysis of high-frequency keywords reveals that these encompass the disease symptoms and associated mechanisms of T2DM and cognitive dysfunction. It is evident that research in this field focuses on the association and interaction between T2DM and cognitive dysfunction. Concurrently, the progression between different degrees of cognitive dysfunction—such as mild cognitive impairment, cognitive impairment, and Alzheimer’s disease—remains a prominent area of scholarly interest. Consequently, exploring the mechanistic linkages between T2DM and cognitive dysfunction, alongside the role of T2DM in the progression of cognitive dysfunction, appears to be a current focal point. Keyword cluster analysis further delineates current research hotspots. The nine clusters identified converge on three primary directions: firstly, the relationship between T2DM and varying degrees of cognitive dysfunction; secondly, the risk factors influencing the disease; and thirdly, relevant therapeutic pathways and mechanisms of action. Among these, cardiovascular disease emerges as a particularly noteworthy risk factor for cognitive dysfunction in T2DM, while functional connectivity and gut microbiota appear to be emerging as novel therapeutic entry points.

Moreover, keyword salience analysis identifies rapidly emerging specialised terminology within short timeframes, thereby revealing deeper developmental shifts. The duration of salience for these keywords exhibits a trend from prolonged to increasingly brief periods. Summarising these salience terms suggests the field’s research can be broadly divided into three phases. Phase One (approximately 2003–2010): Scholars focused on macro-level risks and fundamental pathologies, with research concentrating on vascular risks and Alzheimer’s disease (AD)-related pathologies. Prominent terms during this period included vascular risk factors such as blood pressure, cardiovascular risk factors, and vascular risk factors. Other terms included amyloid precursor protein, atrophy, and white matter lesions, indicating that scholars began exploring the molecular mechanisms linking T2DM to cognitive dysfunction and started examining T2DM’s effects on brain parenchyma. In the second phase (approximately 2011–2018): research into neuroimaging techniques and specific mechanisms deepened. Methods became more refined, focusing on concrete pathogenic factors and more precise brain alterations. Emerging terms included voxel-based morphometry and white matter hyperintensity, signalling the mainstream adoption of advanced neuroimaging techniques to precisely quantify brain atrophy and white matter damage. Severe hypoglycaemia, follow-up, and apolipoprotein E highlighted recognition that clinical treatment side effects constitute significant contributors to cognitive decline, while cohort studies and research into genetic susceptibility gained prominence. Phase Three (2018–2025): Research frontiers shifted towards microscopic mechanisms and systemic perspectives, reflecting contemporary research priorities and emerging trends. The prominence of gut microbiota persisted through 2025, aligning closely with Cluster #3 in the cluster analysis, confirming its status as a current frontier. This also resonates with the gut-brain interaction theory, establishing it as a focal area. The emergence of ‘Mechanisms’ and “stress” indicates recent research is moving beyond observational studies to explore the molecular, cellular, and systems biological mechanisms underlying disease, such as oxidative stress and neuroinflammation. ‘Model,’ as the newest emergent term (2023–2025), may represent researchers’ efforts to construct comprehensive theoretical or animal models to integrate complex pathological pathways and predict disease progression.

Highly cited scholars’ research typically exhibits high quality, providing a robust foundation for studies within their field and reflecting the current state of research. Among these authors, Geert Jan Biessels’ work focuses on dementia risk in diabetic patients (22), though his primary emphasis lies on the mechanisms and clinical significance of cognitive dysfunction in T2DM, yielding valuable findings for the clinical management of this condition (20, 21).José A Luchsinger’s research centres on blood biomarkers in Alzheimer’s disease and diabetes patients, alongside the cognitive impact of vascular disorders (23, 24). Suzanne Craft’s work centres on the mechanisms linking insulin resistance to T2DM and cognitive Alzheimer’s disease (25, 26), while also actively exploring clinical strategies for treating Alzheimer’s disease and cognitive dysfunction (27, 28). The research of these highly cited scholars not only represents the current research foundation and status quo in this field but also guides future research directions. Analysis of co-cited literature reveals that alongside fundamental research into T2DM-related cognitive dysfunction and its mechanisms, scholars also prioritise investigating key risk factors, epidemiological characteristics, and clinical management strategies (25, 29, 30).

### 4.3 Clinical implications and future translation

The bibliometric analysis highlights the multidisciplinary expansion of research on cognitive dysfunction in T2DM, shifting toward microscopic mechanisms (e.g., gut microbiota, oxidative stress) and bridging factors (e.g., functional connectivity). These findings have direct implications for clinical practice. First, regular cognitive screening is recommended, particularly for older T2DM patients (>65 years), using simple tools such as the Mini-Mental State Examination (MMSE), Montreal Cognitive Assessment (MoCA), or Mini-Cog, as endorsed by recent ADA guidelines (2025 update) (31). Early detection of mild cognitive impairment (MCI) could enable timely interventions to slow progression. Second, management strategies should integrate stringent glycemic control, lifestyle modifications (diet, exercise), and comprehensive cardiovascular risk reduction, given the prominence of vascular and metabolic clusters. Emerging evidence supports neuroprotective benefits from antidiabetic agents like GLP-1 receptor agonists (GLP-1RAs) and SGLT2 inhibitors, which may mitigate insulin resistance and inflammation—aligning with recent bursts in ‘mechanisms’ and ‘gut microbiota’. Modulation of gut microbiota (e.g., via probiotics or fecal transplantation) represents a promising translational avenue for improving cognitive outcomes through the gut-brain axis. Third, these bibliometric hotspots could inform updates to clinical guidelines (e.g., IDF or ADA), emphasizing multidimensional risk assessment and personalized approaches to prevent cognitive decline in T2DM patients. Future studies should prioritize translational trials to validate these entry points in clinical settings, bridging the gap between bibliometric trends and bedside application.

## 5 Conclusion

This study employed bibliometric methods to systematically analyse research developments in the field of cognitive dysfunction associated with type 2 diabetes mellitus (T2DM) between 2003 and 2025. Findings indicate vigorous research activity in this domain, characterised by sustained growth in publication output, extensive international collaboration, and leadership from China, the United States, and the United Kingdom, alongside close inter-institutional cooperation. Research focus has progressively expanded from early studies on vascular risk factors and Alzheimer’s disease associations to cutting-edge areas including neuroimaging, gut microbiota, and molecular mechanisms. Current research focuses on the pathological links between T2DM and cognitive dysfunction, associated risk factors, and intervention strategies. Future studies should further integrate multi-omics data, develop disease prediction models, and advance clinical translation to achieve early diagnosis and precision treatment.

## 6 Limitations

A primary limitation of this study is the reliance on the Web of Science Core Collection (WOSCC) as the main data source for bibliometric analysis and calculation of citation metrics (e.g., betweenness centrality). While searches were conducted in PubMed and Scopus to enhance coverage, the final network analyses were performed solely on WOSCC data due to its superior compatibility with CiteSpace and high overlap after deduplication. This may introduce database selection bias, as WOSCC tends to favor English-language journals and certain disciplines, potentially underrepresenting contributions from non-English sources or regions with limited indexing in WoS. For instance, the observed dominance of China in publication output and centrality metrics may partly reflect WOSCC’s stronger coverage of Chinese research in recent years, while underestimating contributions from other non-English-speaking countries. Additionally, citation lag bias may affect the results, as recent publications (particularly those from 2024–2025) have had limited time to accumulate citations, potentially underestimating the impact of emerging research.Self-citation practices could also inflate certain metrics, such as author or institutional centrality, although CiteSpace’s algorithms partially mitigate this through network pruning.

Self-citation practices could also inflate certain metrics, such as author or institutional centrality, although CiteSpace’s algorithms partially mitigate this through network pruning.The analysis is sensitive to CiteSpace parameter settings, including the g-index threshold (k=25) for node selection and pruning methods (pathfinder, pruning sliced networks). Different parameter choices (e.g., lower/higher k values or alternative pruning like minimum spanning tree) may alter cluster structures, burst detection, or centrality rankings, introducing variability in interpretations.Results further depend on the indexing quality and completeness of WOSCC, including potential inconsistencies in author name disambiguation, journal categorization, or missing citations in non-core collections.Finally, this study is purely quantitative and bibliometric in nature, lacking qualitative assessment of individual study design rigor, methodological quality, or reproducibility of the underlying primary research. Future work could complement bibliometric trends with systematic reviews or meta-analyses to evaluate evidence strength.

## Data Availability

The original contributions presented in the study are included in the article/**Supplementary Material**. Further inquiries can be directed to the corresponding author.
