# Supplementary material for: The intellectual landscape of cognitive impairment in type 2 diabetes: knowledge structure, research focuses and rising trends
Source: Front Endocrinol (Lausanne). 2026 Apr 1;17:1809245. doi: 10.3389/fendo.2026.1809245 (PMC13079053; doi:10.3389/fendo.2026.1809245)
Supplement: Supplementary file 1 [file Table1.docx]

Search Strategy for Web of Science

| Rank | Search term | Result |
| --- | --- | --- |
| #1 | TS=(“Diabetes Mellitus, Type 2” OR “Diabetes Mellitus, Non Insulin Dependent” OR “Stable Diabetes Mellitus” OR “Diabetes Mellitus, Type II” OR “NIDDM” OR “Diabetes Mellitus, Noninsulin Dependent” OR “Type 2 Diabetes Mellitus” OR “Noninsulin Dependent Diabetes Mellitus” OR “Type 2 Diabetes” OR “Diabetes, Type 2”) | 220,026 |
| #2 | TS=(“Cognitive Dysfunction” OR “Cognitive Dysfunctions” OR “Dysfunction, Cognitive” OR “Dysfunctions, Cognitive” OR “Cognitive Impairments” OR “Cognitive Impairment” OR “Impairment, Cognitive” OR “Impairments, Cognitive” OR “Cognitive Disorder” OR “Cognitive Disorders” OR “Disorder, Cognitive” OR “Disorders, Cognitive” OR “Mild Cognitive Impairment” OR “Cognitive Impairment, Mild” OR “Cognitive Impairments, Mild” OR “Impairment, Mild Cognitive” OR “Impairments, Mild Cognitive” OR “Mild Cognitive Impairments” OR “Cognitive Decline” OR “Cognitive Declines” OR “Decline, Cognitive” OR “Declines, Cognitive” OR “Mental Deterioration” OR “Deterioration, Mental” OR “Deteriorations, Mental” OR “Mental Deteriorations”) | 193,017 |
| #3 | #1 AND #2 | 3505 |

Search Strategy for PubMed

| Rank | Search term | Result |
| --- | --- | --- |
| #1 | [Title/Abstract] = (“Diabetes Mellitus, Type 2” OR “Diabetes Mellitus, Non Insulin Dependent” OR “Stable Diabetes Mellitus” OR “Diabetes Mellitus, Type II” OR “NIDDM” OR “Diabetes Mellitus, Noninsulin Dependent” OR “Type 2 Diabetes Mellitus” OR “Noninsulin Dependent Diabetes Mellitus” OR “Type 2 Diabetes” OR “Diabetes, Type 2”) | 213,241 |
| #2 | [Title/Abstract] = (“Cognitive Dysfunction” OR “Cognitive Dysfunctions” OR “Dysfunction, Cognitive” OR “Dysfunctions, Cognitive” OR “Cognitive Impairments” OR “Cognitive Impairment” OR “Impairment, Cognitive” OR “Impairments, Cognitive” OR “Cognitive Disorder” OR “Cognitive Disorders” OR “Disorder, Cognitive” OR “Disorders, Cognitive” OR “Mild Cognitive Impairment” OR “Cognitive Impairment, Mild” OR “Cognitive Impairments, Mild” OR “Impairment, Mild Cognitive” OR “Impairments, Mild Cognitive” OR “Mild Cognitive Impairments” OR “Cognitive Decline” OR “Cognitive Declines” OR “Decline, Cognitive” OR “Declines, Cognitive” OR “Mental Deterioration” OR “Deterioration, Mental” OR “Deteriorations, Mental” OR “Mental Deteriorations”) | 174,145 |
| #3 | #1 AND #2 | 2,982 |

Search Strategy for Scopus

| Rank | Search term | Result |
| --- | --- | --- |
| #1 | TITLE-ABS-KEY = (“Diabetes Mellitus, Type 2” OR “Diabetes Mellitus, Non Insulin Dependent” OR “Stable Diabetes Mellitus” OR “Diabetes Mellitus, Type II” OR “NIDDM” OR “Diabetes Mellitus, Noninsulin Dependent” OR “Type 2 Diabetes Mellitus” OR “Noninsulin Dependent Diabetes Mellitus” OR “Type 2 Diabetes” OR “Diabetes, Type 2”) | 309,215 |
| #2 | TITLE-ABS-KEY = (“Cognitive Dysfunction” OR “Cognitive Dysfunctions” OR “Dysfunction, Cognitive” OR “Dysfunctions, Cognitive” OR “Cognitive Impairments” OR “Cognitive Impairment” OR “Impairment, Cognitive” OR “Impairments, Cognitive” OR “Cognitive Disorder” OR “Cognitive Disorders” OR “Disorder, Cognitive” OR “Disorders, Cognitive” OR “Mild Cognitive Impairment” OR “Cognitive Impairment, Mild” OR “Cognitive Impairments, Mild” OR “Impairment, Mild Cognitive” OR “Impairments, Mild Cognitive” OR “Mild Cognitive Impairments” OR “Cognitive Decline” OR “Cognitive Declines” OR “Decline, Cognitive” OR “Declines, Cognitive” OR “Mental Deterioration” OR “Deterioration, Mental” OR “Deteriorations, Mental” OR “Mental Deteriorations”) | 232,748 |
| #3 | #1 AND #2 | 3,523 |
